# Supplementary material for: Larval Therapy and Larval Excretions/Secretions: A Potential Treatment for Biofilm in Chronic Wounds? A Systematic Review
Source: Microorganisms. 2023 Feb 11;11(2):457. doi: 10.3390/microorganisms11020457 (PMC9965881; doi:10.3390/microorganisms11020457)
Supplement: Supplementary file 1 [file microorganisms-11-00457-s001.zip › microorganisms-2100886-supplementary.pdf]

Supplementary Data Table – Table S1: Data extraction table used to qualitatively analyse 12 inclusion criteria meeting research articles

**Title**	**Authors & Ref.**	**Year**	**Extract collection protocol**	**Instar of larvae**	**Extract Quantification**	**Biofilm Assay**	**Isolates characterised**	**Inoculum Quantified**	**Biofilm-forming phenotype confirmed**	**Media**	**Biofilm Incubation Time**	**Monomicrobial/Polymicrobial Biofilm**	**Controls**	**Biofilm Harvesting Method**	**Biofilms Visualised**	**Results Summary**	**Mode of action**
Maggot excretions/secretions are differentially effective against biofilms of Staphylococcus aureus and Pseudomonas aeruginosa	van der Plas *et al.*	2008	5μL H2O per larva for 60 minutes at 35oC	2nd and 3rd Instar	Protein concentration	96-well tissue culture plate	*S. aureus* ATCC 29213<br>*P. aeruginosa* PAO1	No (1:100 dilution for *S. aureus*; 1:1000 for *P. aeruginosa*)	No	0.5 TSB w/0.2% glucose (*S. aureus*)<br>M63 (*P. aeruginosa*)	24 hours	Monomicrobial	Untreated	Solubilisation & Sonication	Not visualised	0.2μg of ES abolished *S. aureus* biofilm formation: 8 hours incubation. Degradation of *P. aeruginosa* biofilms > 10h incubation; required 10-fold more ES than *S. aureus* biofilms. Boiling of ES abrogated their effects on S. aureus, but not on P. aeruginosa biofilms.	Modulation of biofilms by ES did not involve bacterial killing or effects on quorum sensing systems.
Disruption of Staphylococcus epidermidis biofilms by medicinal maggot Lucilia sericata excretions/secretions	Harris *et al.*	2009	200μl H2O per gram of larvae	3rd Instar	Not quantified	96-well tissue culture plate	*S. epidermidis* 1457 (isolated from an infected central venous catheter)<br>*S. epidermidis* 5179 (R1 derived from an infected CSF-shunt)	No (1:100 dilution of overnight cultures used)	Yes	TSB	24 hours	Monomicrobial	Untreated	Solubilisation	Light and immunofluorescence microscopy	In the presence of ES, S. epidermidis 1457 and 5179-R1 nascent biofilm formation was inhibited, and pre-formed biofilms disrupted. ES activity was temperature and time dependent, inactivated by heat treatment, and disruption depended on the mechanism of intercellular adhesion.	ES may contain enzymatic activities, which specifically degrade and functionally inactivate PIA and Aap, respectively.
The Influence of Maggot Excretions on PAO1 Biofilm Formation on Different Biomaterial	Cazander *et al.*	2009	400 Instar 1 or 200 Instar 3 larvae in 100μl 0.9% saline at 35°C in darkness for 1 hour	1st and 3rd Instar	Protein Concentration	96-well tissue culture plate	*P. aeruginosa* PA01	$2.5x10^5$ CFU/mL	Yes	M63	24 hours	Monomicrobial	Untreated	Solubilisation	Not visualised	Maggot ES prevent and inhibit PAO1 biofilm formation and even break down existing biofilms. ES still had considerable biofilm reduction properties after storage at room temperature for 1 month. ES from Instar-3 maggots were more effective than ES from Instar-1 maggots.	N/A
Combinations of maggot excretions/secretions and antibiotics are effective against *Staphylococcus aureus* biofilms and the bacteria derived therefrom	van der Plas *et al*	2010	5μL H2O per larva for 60 minutes at room temperature	2nd and 3rd instar	Protein Concentration	96-well tissue culture plate	*S. aureus* ATCC 29213	No (1:1000 dilution of overnight cultures used)	No	0.5 TSB w/0.2% glucose	24 hours	Monomicrobial	Untreated	Solubilisation	Not visualised	20-200mg/L ES eradicated *S. aureus* biofilms within 3 hours. Enahnced antimicrobial activity of daptomycin against biofilms.	Possibly serine protease related
Maggot excretions inhibit biofilm formation on biomaterials	Cazander *et al.*	2010	400 Instar 1 or 200 Instar 3 larvae in 100μl 0.9% saline at 35°C in darkness for 1 hour	1st and 3rd Instar	Protein concentration	96-well tissue culture plate	*S. aureus*<br>*S. epidermidis*<br>*K. oxytoca*<br>*E. faecalis*<br>*E. cloacae*<br>(prosthetic device isolates)	$2.5x10^5$ CFU/mL 100μl into each well	No	TSB (*S. aureus* & *S. epidermidis*)<br>BHI (*K. oxytoca, E. faecalis* & *E. cloacae*)	3, 5, 7, & 9 days	Monomicrobial	Untreated	Solubilisation	Not visualised	The presence of excretions/secretions reduced biofilm formation on all biomaterials. A maximum of 92% of biofilm reduction was measured.	N/A
Blow fly Lucilia sericata nuclease digests DNA associated with wound slough/eschar and with *Pseudomonas aeruginosa* biofilm	Brown *et al.*	2012	1mL of PBS added to 300 maggots for 30 minutes at room temperature. Maggots left for 1 hour and rinse procedure repeated a further 2 times. 3 in total.	Not stated	Protein Concentration	Lubbock chronic wound pathogenic biofilm (*modified*)	*P. aeruginosa* (Wound isolate)	$1x10^6$ CFU/mL	No	LB Broth	24 hours	Monomicrobial	Untreated	Solubilisation	Confocal microscopy	20μg/mL ES resulted in ~50% reduction in pre-formed biofilms	Investigating a novel Dnase present in larval ES that degraded biofilm structure.

Supplementary Data Table – Table S1: Data extraction table used to qualitatively analyse 12 inclusion criteria meeting research articles

Excretions/secretions from bacteria-pretreated maggot are more effective against *Pseudomonas aeruginosa* biofilms	Jiang *et al.*	2012	50 larvae per 200µl H_2O in	2nd and 3rd instar	Protein Concentration	96-well tissue culture plate	*P. aeruginosa* (Wound isolate)	No (1:100 dilution of overnight cultures used)	No	TSB	24 hours	Monomicrobial	Untreated	Solubilisation	SEM and Brightfield microscopy	Researchers stated that ES obtained from larvae pre-treated with 1x10^6 CFU/mL *P. aeruginosa* displayed enhanced inhibition of nascent biofilm formation.	N/A
Lucilia sericata chymotrypsin disrupts protein adhesin-mediated staphylococcal biofilm formation	Harris *et al*	2013	recombinant Chymotrypsin	Not stated	Protein concentration	96-well tissue culture plate	*S. epidermidis* 1457 (icaADBC and PIA positive) *S. epidermidis* 5179-R1 (Aap positive and icaADBC negative) *S. aureus* SA113 (ATCC 35556 icaADBC, PIA, and SasG positive)	No (1:100 dilution of overnight cultures used)	No	TSB	24 hours	Monomicrobial	Untreated	Solubilisation	Light microscopy	Chymotrypsin derived from maggot excretions/secretions disrupts protein-dependent bacterial biofilm formation mechanisms.	rChymotrypsin proteolytic activity interrupts mechanism for Aap mediated biofilm formation. For pre-formed, rChymotrypsin degrades Aap protein.
Chronic Wounds, Biofilms and Use of Medicinal Larvae	Cowan *et al.*	2013	30 live larvae per assay	Not stated	Not quantified	ex-vivo pig skin explant	*P. aeruginosa* PA01 *S. aureus* SA35556	>10^5 CFU	No	TSA	3 days	Monomicrobial	Untreated	CFU plate count	SEM	After 24 hours of exposure to medical maggots, the levels of both bacteria were 1.7 to 3.3 CFUs per explant, which represents approximately 5-log reduction of total bacteria. After 48 hours of exposure to LDT, no bacterial growth (0 CFUs) was recovered from the processed pig skin explants, indicating total removal of the planktonic and biofilm bacteria.	N/A
Selective Antibiofilm Effects of *Lucilia sericata* Larvae Secretions/Excretions against Wound Pathogens	Bohova *et al.*	2014	Briefly, the third instar nonsterile and sterile larvae were washed and incubated in Milli-Q ultrapure water for 60 min (50 larvae/200µL water) at 4◦ C in a dark place.	3rd Instar	Protein concentration	96-well tissue culture plate	*S. aureus* 1141 *E. cloacae* 2383/10 *P. mirabilis* 719/10 (chronic wound isolates)	10^5 CFU/mL	No	TSB	24 hours	Monomicrobial	Untreated	Solubilisation	Not visualised	Maggot ES at 100 mg/mL concentration significantly reduced biofilm formation thus disrupting established biofilm of *E. cloacae*. Heat-treated ES did not show any antibiofilm activity towards *E. cloacae*. Similar results were obtained in the case of *S. aureus*; however, the heat-treatment of maggot ES did not affect its antibiofilm activity	Antibiofilm fractions contained a protein with MW of around 25 kDa
Antibacterial and anti-biofilm effects of fatty acids extract of dried *Lucilia sericata* larvae against *Staphylococcus aureus* and *Streptococcus pneumoniae* in vitro	Liu *et al*	2021	The fatty acids extract (LFAs) was obtained by mixing 10 g powder with 150 mL 95% ethyl acetate in a 77 ± 2 °C water-bath for 2 h using a fat analyser (BYSXY-06, BY, China), then filtering the solvent to a rotary evaporator,	Not stated	Mass of powder/mL	96-well tissue culture plate	*S. aureus* ATCC 25923 & 29213 *S. pneumoniae* ATCC 6305 & 49619	10^8 CFU/mL	No	TSB w/0.25% glucose	24 hours	Monomicrobial	Untreated	Solubilisation	SEM, TEM and fluorescence microscopy	The amounts of new formed biofilms were gradually decreased with 28.7% - 66.3% and 10.6% - 54.7% as the LFAs concentration rising from 3.9 and 3.1 to 50 and 62.5 µg/mL for *S. aureus* and *S. pneumoniae*, respectively. The total amounts of biofilms were also gradually decreased as the LFAs concentration rising and were less than the initial doses when LFAs' concentrations were more than 62.5 and 50 µg/mL.	Disrupted bacterial membrane and increased memrance permeability.

Supplementary Data Table – Table S1: Data extraction table used to qualitatively analyse 12 inclusion criteria meeting research articles

			and finally fully evaporatin g the solvent														
Maggot Extract Interrupts Bacterial Biofilm Formation and Maturation in Combination with Antibiotics by Reducing the Expression of Virulence Genes	Becerikli *et al.*	2022	Larveel®, a sterile powder made from 100% larvae of *L. sericata*	3rd Instar	Mass of powder/mL	ex-vivo human dermal wound model	*P. aeruginosa* PAO1 *S. aureus* 6850	No	No	DMEM w/ 10% FBS, 1% antibiotics	24 hours	Monomicrobial	Untreated	Coverage before and after on light microscopy images	SEM and light microscopy	Significant reduction in observed biofilms in ex vivo human dermal skin explant model for both bacteria treated.	Determined decreased expression of biofilm maturation and virulence genes in P. aeruginosa after Larveel® treatment
